# IL‐38 prevents induction of trained immunity by inhibition of mTOR signaling

**DOI:** 10.1002/JLB.3A0220-143RRR

**Published:** 2021-02-23

**Authors:** Dennis M. de Graaf, Lisa U. Teufel, Frank L. van de Veerdonk, Leo A. B. Joosten, Mihai G. Netea, Charles A. Dinarello, Rob J. W. Arts

**Affiliations:** ^1^ Department of Medicine University of Colorado Aurora Colorado USA; ^2^ Department of Internal Medicine and Radboud Center for Infectious Diseases Radboud University Medical Center Nijmegen The Netherlands; ^3^ Department of Medical Genetics Iuliu Hatieganu University of Medicine and Pharmacy Cluj‐Napoca Romania; ^4^ Department for Genomics & Immunoregulation Life and Medical Sciences Institute (LIMES) University of Bonn Bonn Germany

**Keywords:** ethanol, inflammation, burn, small RNAs

## Abstract

Trained immunity is the acquisition of a hyperresponsive phenotype by innate immune cells (such as monocytes and macrophages) after an infection or vaccination, a *de facto* nonspecific memory dependent on epigenetic and metabolic reprogramming of these cells. We have recently shown that induction of trained immunity is dependent on IL‐1β. Here, we show that recombinant IL‐38, an anti‐inflammatory cytokine of the IL‐1‐family, was able to induce long‐term inhibitory changes and reduce the induction of trained immunity by β‐glucan in vivo in C57BL/6 mice and *ex vivo* in their bone marrow cells. IL‐38 blocked mTOR signaling and prevented the epigenetic and metabolic changes induced by β‐glucan. In healthy subjects, the *IL1F10* associated single nucleotide polymorphism rs58965312 correlated with higher plasma IL‐38 concentrations and reduced induction of trained immunity by β‐glucan *ex vivo*. These results indicate that IL‐38 induces long‐term anti‐inflammatory changes and also inhibits the induction of trained immunity. Recombinant IL‐38 could therefore potentially be used as a therapeutic intervention for diseases characterized by exacerbated trained immunity.

## Graphical Abstract

IL‐38 inhibits the induction of innate immune memory

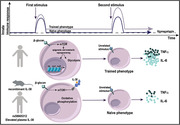

## 1 INTRODUCTION

An increasing number of studies have shown that monocytes and macrophages can acquire long‐term functional adaptive changes, a de facto innate memory that has been termed “trained immunity.”^1^, ^2^, ^3^ The functional remodeling of myeloid cells is mediated by epigenetic and metabolic rewiring, including of their bone marrow progenitors.^4^, ^5^, ^6^, ^7^ Trained immunity can be induced by vaccines such as bacillus Calmette‐Guerin (BCG) that protects against *Mycobacterium tuberculosis*, or immunomodulators such as β‐glucan, a common fungal cell wall component.^8^, ^9^ The rewiring of the epigenome of myeloid cells often results in long‐lasting modifications such as increased host defense to unrelated infections.^1^, ^10^ For example, BCG vaccination of neonates in West Africa reduced mortality from infections,^11^ and BCG vaccination in healthy adults reduced subsequent yellow fever vaccine viremia and parasitemia in a model of human experimental malaria, which correlated with the extent of BCG‐dependent epigenetic changes in circulating monocytes.^8^, ^12^ In mouse models in which trained immunity is induced with *Candida albicans* derived β‐glucan, an enhanced proinflammatory state is reached, which results in reduced mortality from, for example, fungal and *Staphylococcus aureus* sepsis.^7^, ^9^

The IL‐1 family consists of 11 members^13^, ^14^; amongst which IL‐37 and IL‐38 have broad anti‐inflammatory properties.^15^, ^16^ Previously, we have reported that IL‐1β is central in the induction of trained immunity^12^ and that IL‐37 inhibits the induction of trained immunity.^17^ Furthermore, IL‐1β was found to be the cytokine most significantly increased in bone marrow of β‐glucan‐treated mice and causative for the induction of trained immunity.^7^ In in vitro models of trained immunity, primary human monocytes respond to IL‐1β with epigenetic changes that augment the production of inflammatory cytokines such as TNFα and IL‐6.^12^ Furthermore, the A allele of the single nucleotide polymorphism (SNP) rs16944 in the promotor region of the *IL1B* gene was associated with increased induction of trained immunity in human monocytes.^12^

We have previously reported that IL‐38 can inhibit IL‐1β, IL‐6, and KC in a model of gouty arthritis.^18^ As IL‐38 can block IL‐1β, we hypothesize that IL‐38 has an inhibitory role for the induction of trained immunity. In this report, we aimed to define the role of IL‐38 in β‐glucan‐induced trained immunity. We show that IL‐38 inhibits the induction of trained immunity in murine models by disrupting the mTOR signaling cascade, which is essential for the induction of trained immunity.^5^ In healthy humans, we show that the SNP rs58965312 affects IL‐38 plasma levels and the trained immunity response in vitro.

## 2 MATERIALS AND METHODS

### 2.1 Mice

Seven to nine‐week‐old male C57Bl/6 mice were obtained from Jackson Laboratories (Bar Harbor, ME, USA). The mice were fed sterilized laboratory chow and water ad libitum. The experiments were approved by the Institutional Animal Care and Use Committees of the University of Colorado Denver (Aurora, CO, USA).

### 2.2 Trained immunity model

Mice received 1 μg of bioactive^18^, ^19^ human recombinant IL‐38 (3‐152) (Biotechne, Minneapolis, MN, USA) i.p. in sterile saline in a 200 μl volume or only saline for three consecutive days. On the first day, 1 h after injection of IL‐38, mice received i.p. A total of 1 mg of β‐glucan (β‐1,3‐(D)‐glucan) kindly provided by Professor David Williams (College of Medicine, Johnson City, TN, USA) or 200 μl saline as vehicle control. On the fifth day, mice were directly sacrificed or first challenged with 5 mg/kg LPS (*Escherichia coli* [055:B5] Sigma‐Aldrich, St. Louis, MO, USA) i.p. in 200 μl sterile saline 4 h before sacrifice. Mouse body temperature was measured with a MT4 thermometer (RayTek MiniTemp, Wilmington, NC, USA). After anesthetization with isoflurane, blood from the orbital plexus was collected in EDTA, and mice were sacrificed by cervical dislocation. Blood was centrifuged at 1000 ×*g* for 10 min to prepare plasma for cytokine measurements.

### 2.3 In vitro cytokine production

Cytokine production was assessed *ex vivo* in whole blood, bone marrow cells, and splenocytes.

Whole blood was diluted 1:4 times in RPMI 1640. A total of 200 μl volume was plated in 96‐well round bottom plates and incubated for 24 h with no stimulus or 100 ng/ml LPS (±10 μM Nigericin [Invivogen, San Diego, CA, USA] during the last hour) at 37°C in 5% CO_2_. After 24 h, 180 μl of supernatant was collected and the cell pellet was stored in 0.5% Triton X‐100 (Sigma, St. Louis, MO, USA). Femoral bone marrow was suspended in RPMI culture medium, counted on HemaTrue cell counter (Heska, Loveland, CO, USA) and adjusted to 1 × 10^6^/ml. A total of 200 μl of the cell suspension was plated in 96‐well round bottom plates and incubated for 24 h in RPMI (no growth factors added) in same conditions as described earlier. Spleens were aseptically removed, homogenized through 100 μm cell strainers (Thermo Fisher Scientific, Waltham, MA, USA), and collected in RPMI. Cells were counted and adjusted to 5 × 10^6^/ml. A total of 200 μl of the cell suspension was plated in 96‐well round bottom plates and incubated for 24 h or 3 d in RPMI containing 10% FBS in same conditions as described earlier.

### 2.4 Cytokine measurement by ELISA and lactate measurement

Supernatants and plasma were stored at −20°C until further analysis. ELISA was performed according to the manufacturer's instructions (BioTechne), except the IL‐38 ELISA on human plasma was performed as described elsewhere.^20^

Mouse plasma and bone marrow were obtained as described and lactate levels were determined by a commercially available Lactate Fluorometric Assay Kit (Biovision, Milpitas, CA, USA).

### 2.5 Western blot

Bone marrow from naïve mice was collected as described earlier. A total of 1 × 10^6^ cells were cultured in a 12‐well flat‐bottom plate and incubated for 3 d in RPMI containing 10% serum. Supernatant with nonadherent cells was removed. After 4 h incubation (which was optimal for mTOR phosphorylation) with or without 5 μg/ml β‐glucan ± 100 ng/ml IL‐38 in RPMI, cells were lysed in RIPA buffer containing protease inhibitor (Roche, Basel, Switzerland). Total protein content was determined by BCA assay (Thermo Fisher Scientific), and equal amounts of proteins were subjected to SDS‐PAGE on precasted 4–15% gels (Biorad, Hercules, CA, USA). The separated proteins were transferred to a nitrocellulose membrane (Biorad), which was blocked in 5% BSA (Sigma) and incubated overnight at 4°C with rabbit polyclonal antibodies against (phospho) mTOR, Akt, 4EBP1, and S6K (Cell Signaling, Danvers, MA, USA), and β‐actin (Sigma), which were visualized using a polyclonal secondary antibody (Dako, Leuven, Belgium) and SuperSignal West Femto Substrate (Thermo Fisher Scientific) for the phosphorylated proteins or ECL substrate for the other proteins (Biorad).

### 2.6 mRNA extraction and RT‐PCR

mRNA from mouse bone marrow was isolated with Trizol (Life Technologies, Carlsbad, CA, USA) chloroform (Sigma) extraction, followed by isopropanol precipitation (Sigma) and two washes with 70% ethanol (Sigma). cDNA was synthesized using iScript reverse transcriptase (Invitrogen, Carlsbad, CA, USA). Relative expression was determined using the SYBR Green method (Invitrogen) on a CFX96 Bio‐Rad qPCR machine (Biorad), and the values were expressed as fold increases in mRNA levels, relative to vehicle control mice, with B2M as a housekeeping gene. Primers are listed in Supporting Information Table S1.

### 2.7 Chromatin immunoprecipitation

Bone marrow was isolated as described earlier. Cells were fixed in 1% methanol‐free formaldehyde and stored at 4°C. Afterward, cells were sonicated, and immunoprecipitation was performed using antibodies against H3K4me3 (Diagenode, Seraing, Belgium), as described elsewhere.^6^ DNA was isolated with a MinElute PCR purification kit (Qiagen, Germantown, MD, USA) and was further processed for qPCR analysis using the SYBR green method on a Step‐one PLUS qPCR machine (Applied Biosciences, Foster City, CA, USA). Samples were analyzed by a comparative Ct method according to the manufacturer's instructions. The epigenetics primers are listed in Supporting Information Table S1.

### 2.8 PBMC experiments

The study was performed in a cohort of ±200 healthy individuals of Western European ancestry from the Human Functional Genomics Project (200FG cohorts, see www.humanfunctionalgenomics.org). PBMCs were isolated from consenting healthy donors as described before.^21^ The preparation of plasma for IL‐38 ELISA and in vitro stimulations of primary monocyte stimulations in this cohort were described elsewhere.^22^ Monocytes were trained with β‐glucan for 24 h, washed with warm PBS, and rested in RPMI for 6 d.^23^ Then, cells were stimulated with 10 ng/ml LPS. After 24 h, supernatants were collected and stored at −20°C until IL‐6 and TNFα were measured by ELISA. Plasma IL‐38 levels were determined by ELISA. The status of IL‐38 SNP rs58965312 was assessed as described in the methods of Li et al.^22^ by the commercially available SNP chip, Illumina HumanOmniExpressExome‐8 v1.0.

### 2.9 Statistical analysis

The differences between the various conditions were analyzed with the Wilcoxon matched pairs signed rank test or Mann‐Whitney *U*‐test as appropriate. Data are presented as mean ± sem unless otherwise indicated. Data were analyzed using GraphPad Prism 8.0 (GraphPad Software, La Jolla, CA, USA). A *P*‐value below 0.05 was considered significant.

## 3 RESULTS

### 3.1 IL‐38 inhibits LPS‐induced inflammation

**FIGURE 1:**
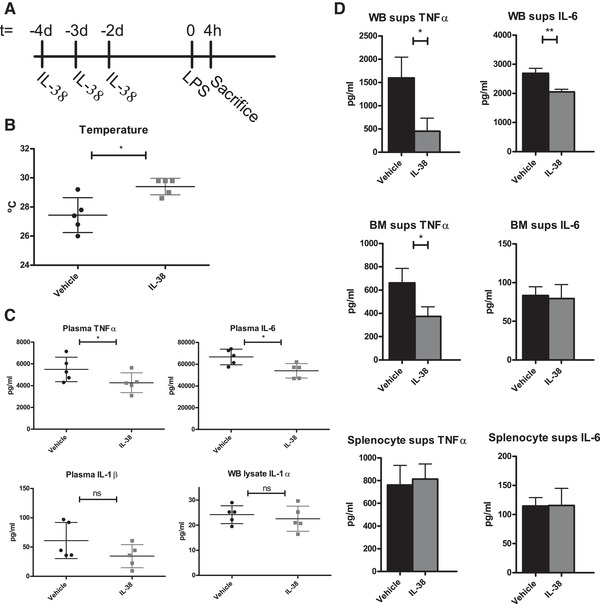
**Effect of IL‐38 on the inflammatory response to LPS**. (**A**) Experimental outline. Mice received on three consecutive days 1 μg IL‐38 i.p. or vehicle. All mice received on day 5 mg/kg LPS i.p. and were sacrificed 4 h later. (**B**) Abdominal wall temperature of mice at sacrifice. (**C**) Cytokine levels in plasma and blood lysate at sacrifice. (**D**) Spontaneous TNFα and IL‐6 production in supernatants from whole blood, bone marrow, and splenocytes after 24 h *ex vivo* culture. *N* = 5, mean ± sem, cytokine production normalized to monocyte numbers *P < 0.05, **P < 0.01, ns not significant

These studies on the putative role for IL‐38 on trained immunity began with an assessment of the effect of recombinant human IL‐38 on systemic inflammation due to endotoxemia. Mice received either vehicle or 1 μg of IL‐38 i.p. on days 4, 3, and 2 (Fig. 1A). After 4 d, LPS (5 mg/kg) was administered i.p. to all mice, and 4 h later the mice were sacrificed. As shown in Figure 1B, mice receiving IL‐38 exhibited less hypothermia compared to vehicle treated mice, indicating reduced inflammation.^24^ Plasma IL‐1β, TNFα and IL‐6 concentrations were significantly decreased in mice treated with recombinant IL‐38 compared to vehicle‐treated mice (Fig. 1C), whereas IL‐1α concentration in lysates of whole blood was not affected by IL‐38. In contrast, no major IL‐38‐dependent changes in cell counts in bone marrow, splenocytes, and whole blood were observed, apart from a small decrease in lymphocytes in whole blood (data not shown). Whole blood, bone marrow, and splenocytes were further incubated in vitro for 24 h without any additional stimulus. We observed reduced spontaneous release of TNFα (75% decrease) and IL‐6 (25% decrease) from whole blood cultures of mice treated with IL‐38 (Fig. 1D top). In cultured bone marrow, a reduced spontaneous release of TNFα (55% decrease) was also observed (Fig. 1D middle). Splenocytes did not show differences in IL‐6 or TNFα production (Fig. 1D bottom).

### 3.2 IL‐38 inhibits β‐glucan‐induced trained immunity

**FIGURE 2:**
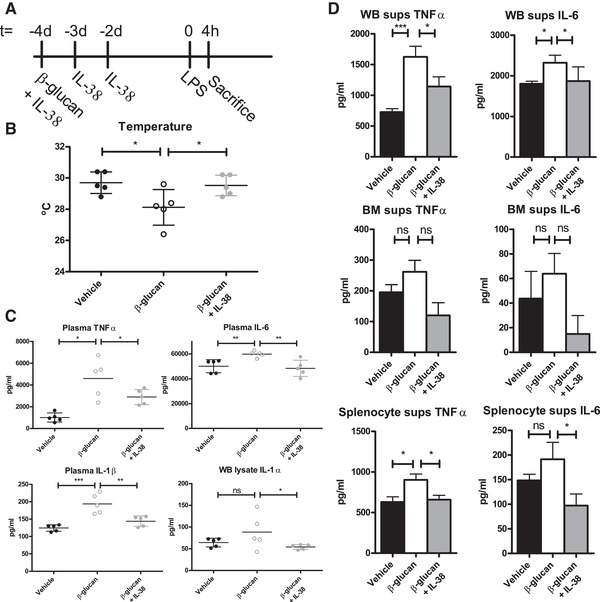
**Effect of IL‐38 on the induction of trained immunity by β‐glucan**. (**A**) The experimental outline is comparable to Figure 1, but now after the first dose of IL‐38, 1 mg of β‐glucan (or vehicle) was administered i.p. (**B**) Temperature at sacrifice. (**C**) Cytokine levels in plasma and blood lysate at sacrifice. (**D**) Spontaneous TNFα and IL‐6 production in supernatants from whole blood, bone marrow, and splenocytes after 24 h *ex vivo* culture. *N* = 5, mean ± sem, cytokine production normalized to monocyte numbers *P < 0.05, **P < 0.01, ***P < 0.001, ns not significant

Next, we assessed whether IL‐38 was also able to inhibit the induction of trained immunity by β‐glucan. β‐glucan‐induced trained immunity results in amplified cytokine production to a secondary and unrelated challenge.^7^, ^9^ As depicted in Figure 2A, we administered recombinant IL‐38 (1 μg) or saline to mice 1 h before i.p. β‐glucan (1 mg) on day ‐4. On days ‐3 and ‐2, mice received 1 μg of IL‐38 per day. After 2 d of rest, all mice received 5 mg of LPS. Four hours after LPS administration, the abdominal wall temperature of mice treated with β‐glucan was reduced by 2°C compared to mice treated with vehicle and normalized by treatment with IL‐38 (Fig. 2B). In mice treated with β‐glucan and then challenged with LPS, a similar reversal of the effects of β‐glucan by IL‐38 was observed in plasma TNFα, IL‐6, and IL‐1β (Fig. 2C). As expected in *ex vivo* cultured whole blood (without extra *ex vivo* stimulation) from β‐glucan‐trained mice an increase in TNFα and IL‐6 production was found, which was abrogated by the addition of IL‐38 (Fig. 2D). This cytokine profile was reflected in splenocyte cultures and—although not statistically significantly—in bone marrow (Fig. 2D).

### 3.3 IL‐38 prevents epigenetic histone changes induced by β‐glucan

**FIGURE 3:**
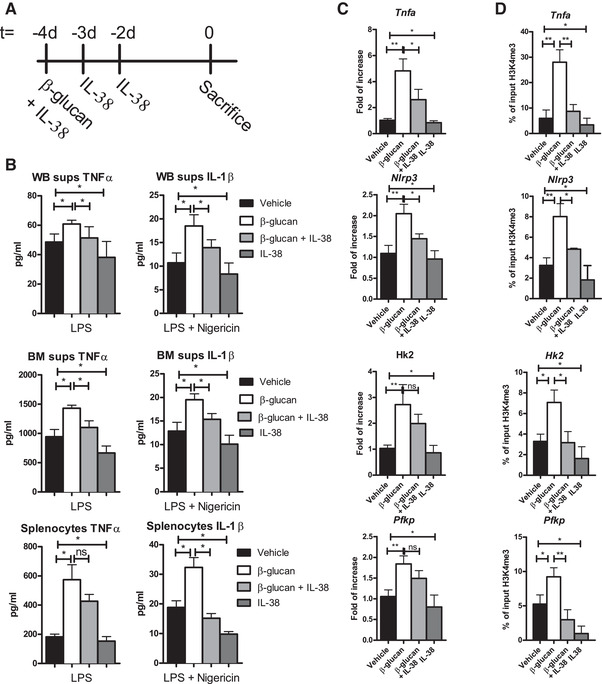
**Epigenetic effect of IL‐38 on the induction of trained immunity by β‐glucan**. (**A**) The experimental outline is comparable to Figure 2, but now no LPS administration (except for panel C). (**B**) TNFα and IL‐1β production in supernatants from whole blood, bone marrow, and splenocytes after 24 h culture with 100 ng/ml LPS and 1 h 10 μM Nigericin. (**C**) RNA expression in bone marrow after 4 h in vivo LPS. (**D**) Percentage of H3K4me3 at the promoters of genes displayed. *N* = 12, mean ± sem, cytokine production normalized to monocyte numbers, *P < 0.05, **P < 0.01, ns not significant

As the heightened induction of inflammatory cytokines by β‐glucan is the result of epigenetic changes at the level of histones,^4^, ^9^ we next assessed the influence of IL‐38 on epigenetic modifications at promoters of established trained immunity‐related genes. Mice were treated with β‐glucan ± IL‐38 and sacrificed after 5 d without LPS challenge (Fig. 3A).^2^, ^6^ When whole blood, bone marrow, and splenocytes were stimulated *ex vivo* with LPS with or without the inflammasome activator nigericin, an increase in cytokine production was seen in β‐glucan trained mice, which was abolished by IL‐38 (Fig. 3B). When expression of genes related to trained immunity (*Tnfa*, *Nrlp3*, *Hk2*, and *Pfkp*) was determined in bone marrow of LPS‐challenged mice, increased expression of all genes was observed which was prevented by IL‐38 (Fig. 3C). To determine whether this is the result of epigenetic modifications induced by β‐glucan and IL‐38, bone marrow of non‐LPS treated mice was processed for epigenetic analysis. H3K4me3 (an important histone marker in trained immunity^4^, ^9^) marked DNA was isolated, and the percentage of promoters of the trained immunity‐related genes that were positive for this histone mark was determined.^2^ As shown earlier, an increase in promotors positive for H3K4me3 was seen in β‐glucan‐trained mice, and this was reversed when mice also received IL‐38 (Fig. 3D).

### 3.4 IL‐38 obstructs β‐glucan dependent Akt/mTOR/S6K signaling

**FIGURE 4:**
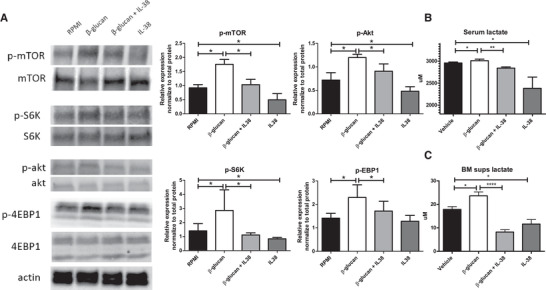
**Effect of IL‐38 on β‐glucan‐dependent phosphorylation of the mTOR pathway**. (**A**) After naïve bone marrow incubation with IL‐38 (100 ng/ml) or vehicle for 30 min and subsequent β‐glucan (5 μg/ml) or vehicle for 4 h Western blot for indicated proteins was performed on the cell lysate. Actin and total proteins were used as loading control. The graphs represent the phosphorylated protein divided by the total protein in arbitrary units, *N* = 3 (**B**) Mice were treated following the protocol of Figure 2A. Four hours after LPS stimulation serum lactate levels were determined, and (**C**) bone marrow was isolated and plated for 72 h after which lactate levels were determined in the supernatant. *N* = 5 (B‐C), mean ± sem, *P < 0.05, **P < 0.01, ****P < 0.0001, ns not significant

We have shown previously that trained immunity in monocytes depends on activation of the mTOR pathway.^5^ As IL‐38 inhibits the induction of trained immunity, we hypothesized that inhibition of the mTOR pathway takes place, as was also the mechanism for IL‐37 inhibition of trained immunity.^17^ As shown in Figure 4A, the induction of trained immunity in bone marrow from naïve mice by β‐glucan in vitro induces phosphorylation of Akt, mTOR, and its downstream kinases S6K and 4EBP1. IL‐38 reduced this phosphorylation, indicating that IL‐38 inhibits the activation of this pathway.

As mTOR is a central regulator of cellular metabolism, we determined the activity of the glycolysis pathway, which is highly induced in β‐glucan trained monocytes and macrophages.^5^ As the end product of anaerobic glycolysis, lactate levels were measured. IL‐38 treatment prevented the increase of lactate levels in plasma 4 h after treatment with LPS and in supernatants of 72 h cultured bone marrow from mice trained with β‐glucan and injected with LPS in vivo (Fig. 4B, C).

### 3.5 SNP and IL‐38 plasma levels predict the strength of trained immunity induction

**FIGURE 5:**
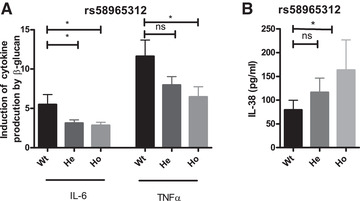
**Effect of IL‐38 (*IL‐1F10*) single nucleotide polymorphism (SNP) rs58965312 on β‐glucan training response and plasma IL‐38**. (**A**) Monocytes were trained with β‐glucan or control for 24 h. One week later, cells were stimulated for 24 h with 10 ng/ml LPS and IL‐6 and TNFα were determined in supernatant. Induction of cytokine production (β‐glucan/control) was determined. (**B**) Plasma levels of IL‐38 in corresponding individuals. Wt = CC = 35, He = CT = 53, Ho = TT = 30, mean ± sem, *P < 0.05, ns not significant

To validate the influence of IL‐38 on trained immunity in humans, we investigated the effect of 11 SNPs associated with IL‐38 (*IL1F10*) and the adjacent IL‐1Ra (*IL‐1RN*) on the induction of trained immunity in human PBMC from 118 healthy subjects of the 200FG cohort.^25^ We measured plasma IL‐38 concentrations, and we quantified the capacity of their monocytes to acquire trained immunity. As shown in Figure 5A, the induction of IL‐6 and TNFα after in vitro training with β‐glucan is related to the SNP rs5896312. In addition, the plasma level of IL‐38 was dependent on this SNP, as carriers of two alternative alleles had higher levels in comparison to subjects carrying two reference alleles (Fig. 5B). No difference was found in plasma IL‐1RA levels of these individuals (data not shown). In summary, the inhibition of trained immunity in healthy volunteers was associated with a higher concentration of plasma IL‐38, which in turn is dependent on the SNP rs5896312.

## 4 DISCUSSION

In this report we demonstrate that IL‐38 significantly reduces the induction of trained immunity by β‐glucan. This observation is consistent with the anti‐inflammatory properties of IL‐38 as an inhibitor of innate and adaptive immunity.^16^, ^25^ When mice are trained with β‐glucan, epigenetic modifications occur in cells of the innate immune system, resulting in enhanced proinflammatory responses of the innate immune system upon a second inflammatory challenge. Enhanced production of proinflammatory cytokines also results in epigenetic modifications in the bone marrow,^7^ and reduces mortality in models of systemic *C. albicans* or *S. aureus* infections.^8^, ^9^ Moreover, IL‐1β has been shown to play a major role in the induction of trained immunity,^7^, ^12^ which is also highlighted by Van der Meer et al., who demonstrated that treating mice with small doses of recombinant human IL‐1β reduces mortality in granulocytopenic mice from subsequent *Pseudomonas* infection, a concept at that time termed nonspecific resistance to infection.^27^ Another member of the IL‐1 family, IL‐37, was shown to inhibit the NLRP3 inflammasome and the release of active IL‐1β, and the induction of trained immunity.^17^ Given the anti‐inflammatory properties of IL‐38,^28^ such as its ability to block IL‐1β upregulation in mouse models of gouty arthritis,^18^ we investigated whether IL‐38 can block the induction of trained immunity.

Here, we show that in addition to preventing LPS‐induced inflammation in mice,^29^ IL‐38 abrogated the induction of trained immunity and the subsequent response to secondary stimulus with LPS. IL‐38 administration inhibited β‐glucan‐induced phosphorylation of the Akt/mTOR signaling pathway. Similarly, IL‐38 prevented β‐glucan dependent proinflammatory epigenetic reprogramming, as shown by a reduction in H3K4me3 on promotors of *Tnfa*, *Nrlp3*, *Hk2*, and *Pfkp* in mouse bone marrow. In vivo treatment with β‐glucan and IL‐38 and subsequent LPS administration resulted in lower concentrations of lactate in plasma and in *ex vivo* cultured bone marrow supernatants. Lower concentrations of lactate indicate that IL‐38 blocks glycolysis, most likely through inhibition of the mTOR pathway. These observations are confirmed by analysis of a cohort of healthy subjects in which the SNP rs5896312 adjacent to the gene encoding IL‐38 affected both plasma IL‐38 levels and the inducibility of trained immunity in monocytes *ex vivo*. Taken together, we show that a reduced level of IL‐38 results in increased induction of trained immunity, whereas administration of recombinant IL‐38 inhibits this biological response.

IL‐38 binds to the IL‐36R, and IL‐1R9 is its putative coreceptor.^16^, ^30^ It has been reported that N‐terminally truncated IL‐38 attenuates the JNK/AP1 pathway which results in reduced IL‐6 production from macrophages.^30^ Here we show that IL‐38 also inhibits the Akt/mTOR pathway, which is essential in the induction of trained immunity.^5^ Interestingly, inhibition of the mTOR pathway has also been observed for IL‐37,^31^, ^32^ which suggests a partially comparable working mechanism of these broad anti‐inflammatory IL‐1 family members.

Besides the positive role of trained immunity in providing protection against infection, a deleterious effect has been suggested in auto‐immune diseases.^3^ Therefore, IL‐38 may be used therapeutically to temper trained immunity in these diseases. Trained immunity was specifically suggested to play a pathologic role in systemic lupus erythematosus (SLE), Sjögren's syndrome and rheumatoid arthritis.^3^ A protective role of IL‐38 was proposed in SLE,^26^ in mice subjected to experimental arthritis,^33^ and in Sjögren's disease IL‐38 was up‐regulated as a counterbalance to IL‐36‐dependent inflammation.^34^ Hence, a novel hypothesis is that recombinant IL‐38 could be used to inhibit inflammation in auto‐immune diseases by inhibiting trained immunity.

Trained immunity was also suggested to play a role in auto‐inflammatory diseases. We have previously reported that in patients with hyper IgD syndrome, the deficiency of mevalonate kinase results in the accumulation of mevalonate, and an overactive trained immunity phenotype presents as attacks of sterile inflammation. To date, there are limited data on the role of IL‐38 in IL‐1β or NLRP3 related auto‐inflammatory syndromes. The inhibition of IL‐1β by IL‐38 and the homology with IL‐1Ra warrants further investigation on the effects of IL‐38 in these diseases and in particular on the assembly of the inflammasome and IL‐1β processing, as an anti‐inflammatory role for IL‐38 in these IL‐1β‐driven diseases could be hypothesized. We currently do not know which receptor pathway is blocked by IL‐38 that results in a reduction of IL‐1β signaling. We consider that IL‐38 can interfere with IL‐36R signaling, which can bind IL‐36 cytokines, and induces IL‐17 and IL‐1β. Additionally, IL‐38 may interfere with IL‐1R9 which is also involved in inducing IL‐17.^35^ IL‐1β itself can induce IL‐36 cytokines and IL‐17, resulting in an auto‐inflammatory loop that can be broken by IL‐38. However, most importantly we show that IL‐38 inhibits the mTOR pathway, elucidating one of the mechanisms that IL‐38 uses to inhibit IL‐1β production.

This study presents evidence that IL‐38 inhibits the induction of trained immunity by interfering with the mTOR pathway and with IL‐1 signaling. Apart from interfering with these essential signaling pathways in the induction of trained immunity by β‐glucan, IL‐38 itself also induced epigenetic changes, which suggests that separate from inhibiting trained immunity IL‐38 might have a long‐term immunosuppressive effect. The role of IL‐38 in human diseases that depend on trained immunity requires further characterization, for example by assessment of human samples to determine the effect of IL‐38 on inflammation in these diseases’ contexts. The metabolic effects of IL‐38 and its effects on trained immunity become especially relevant in the setting of cardiovascular disease. Trained immunity is thought to contribute to persistent inflammation that characterizes atherosclerosis.^36^ One marker of this persistent inflammation is C‐reactive protein (CRP) and in a meta‐analysis of more than 80,000 subjects there were polymorphisms found in only five immune genes that associated with circulating CRP, which included *CRP* itself, *NRLP3*, *IL‐6R*, and strikingly *IL‐1F10* (IL‐38).^37^ We reported recently that IL‐38 plasma concentrations correlate inversely with IL‐6 and CRP in overweight subjects, and are most reduced in subjects with metabolic syndrome.^20^ These and our current observations suggest that IL‐38 might be a key player in restraining metabolic pathways and inflammation that are crucial drivers of cardiovascular disease.

In conclusion, in this study we show that IL‐38 is an important anti‐inflammatory mediator that inhibits the induction of trained immunity. IL‐38 inhibits the induction of epigenetic and metabolic changes induced by β‐glucan, and a SNP that correlates with higher IL‐38 concentrations in healthy volunteers results in reduced induction of trained immunity by β‐glucan *ex vivo*. These data support the hypothesis that modulation of IL‐38 could be of therapeutic use in diseases in which trained immunity is dysregulated and contributes to disease pathology.

## AUTHORSHIP

D.M.G., M.G.N., C.A.D., and R.J.W.A. designed the experiments. D.M.G., L.U.T., C.A.D., and R.J.W.A. performed the experiments. F.L.V., L.A.B.J., and M.G.N. contributed clinical samples. L.A.B.J., M.G.N., and C.A.D. contributed reagents/materials. D.M.G. and R.J.W.A. wrote/drafted and finalized the paper. All authors read and approved the final manuscript.

## DISCLOSURES

The authors declare no conflicts of interest.

## Supporting information

Table 1. Primer sequences.
