# Supplementary material for: IL‐38 prevents induction of trained immunity by inhibition of mTOR signaling
Source: J Leukoc Biol. 2021 Feb 23;110(5):907–15. doi: 10.1002/JLB.3A0220-143RRR (PMC8380748; doi:10.1002/JLB.3A0220-143RRR)
Supplement: Supplementary file 1 — Table 1. Primer sequences. [file JLB-110-907-s001.docx]

**Table 1. Primer sequences.**

| qPCR Primers | | |
| --- | --- | --- |
| *Tnfa* | 5'>3' | CAGACCCTCACACTCAGATCATCT |
|  | 3'>5' | CCTCCACTTGGTGGTTTGCTA |
| *Nlrp3* | 5'>3' | ATCAACAGGCGAGACCTCTG |
|  | 3'>5' | GTCCTCCTGGCATACCATAGA |
| *Pfkpf* | 5'>3' | GAAACATGAGGCGTTCTGTGT |
|  | 3'>5' | CCCGGCACATTGTTGGAGA |
| *Hk2f* | 5'>3' | TGATCGCCTGCTTATTCACGG |
|  | 3'>5' | AACCGCCTAGAAATCTCCAGA |
| Epigenetic primers | | |
| *Tnf* | 5'>3' | CTTGGGCCAGTGAGTGAAAG |
|  | 3'>5' | TAGCCAGGAGGGAGAACAGA |
| *Nlrp3* | 5'>3' | GGGACCAAATTGAGGGCTTC |
|  | 3'>5' | TCAACGTCACCAGTCCTCAGA |
| *Pfkpf* | 5'>3' | ACCGATAGCTTTGCCATCCC |
|  | 3'>5' | TCTGGCGTCTCTACCTCCTC |
| *Hk2f* | 5'>3' | AGCTGAGGGCCTCAAGTTTC |
|  | 3'>5' | CTAAGCAGCTAGACCGGTCG |
